# Overexpression of the *NMig1* Gene Encoding a NudC Domain Protein Enhances Root Growth and Abiotic Stress Tolerance in *Arabidopsis thaliana*

**DOI:** 10.3389/fpls.2020.00815

**Published:** 2020-06-11

**Authors:** Valentin Velinov, Irina Vaseva, Grigor Zehirov, Miroslava Zhiponova, Mariana Georgieva, Nick Vangheluwe, Tom Beeckman, Valya Vassileva

**Affiliations:** ^1^Department of Molecular Biology and Genetics, Institute of Plant Physiology and Genetics, Bulgarian Academy of Sciences, Sofia, Bulgaria; ^2^Department of Plant Physiology, Faculty of Biology, Sofia University “St. Kliment Ohridski”, Sofia, Bulgaria; ^3^Department of Plant Biotechnology and Bioinformatics, Ghent University, Ghent, Belgium; ^4^VIB-UGent Center for Plant Systems Biology, Ghent, Belgium

**Keywords:** *NudC* gene, *NMig1*, abiotic stress, reactive oxygen species, heat shock proteins, *Arabidopsis thaliana*

## Abstract

The family of NudC proteins has representatives in all eukaryotes and plays essential evolutionarily conserved roles in many aspects of organismal development and stress response, including nuclear migration, cell division, folding and stabilization of other proteins. This study investigates an undescribed *Arabidopsis* homolog of the *Aspergillus nidulans NudC* gene, named *NMig1* (for *Nuclear Migration 1*), which shares high sequence similarity to other plant and mammalian *NudC*-like genes. Expression of *NMig1* was highly upregulated in response to several abiotic stress factors, such as heat shock, drought and high salinity. Constitutive overexpression of *NMig1* led to enhanced root growth and lateral root development under optimal and stress conditions. Exposure to abiotic stress resulted in relatively weaker inhibition of root length and branching in *NMig1-*overexpressing plants, compared to the wild-type Col-0. The expression level of antioxidant enzyme-encoding genes and other stress-associated genes was considerably induced in the transgenic plants. The increased expression of the major antioxidant enzymes and greater antioxidant potential correlated well with the lower levels of reactive oxygen species (ROS) and lower lipid peroxidation. In addition, the overexpression of *NMig1* was associated with strong upregulation of genes encoding heat shock proteins and abiotic stress-associated genes. Therefore, our data demonstrate that the *NudC* homolog *NMig1* could be considered as a potentially important target gene for further use, including breeding more resilient crops with improved root architecture under abiotic stress.

## Introduction

*Nuclear distribution C* (*NudC*) genes encode proteins with high structural and functional conservation from yeast to human (Osmani et al., 1990; Fu et al., 2016). *NudC* has been identified in the filamentous fungus *Aspergillus nidulans* as an essential gene required for the microtubule-dependent migration of cell nuclei and for normal colony growth (Osmani et al., 1990; Chiu et al., 1997). NudC proteins play roles in diverse cellular processes, including cell division, cell cycle progression, neuronal migration, dynein-dependent functions (Aumais et al., 2003; Zhou et al., 2003; Fu et al., 2016). Homologs of NudC from mammals, Drosophila, *Caenorhabditis elegans* and *Arabidopsis thaliana* complement *nudC3* mutation in *A. nidulans*, and result in the normal movement of nuclei and colony growth (Cunniff et al., 1997; Morris et al., 1997; Dawe et al., 2001). These observations suggest that some functions of NudC are evolutionarily and functionally conserved in different eukaryotic organisms.

The levels of NudC mRNA and protein correlate with the proliferative activity in different cell types and tissues. The human NudC homolog is highly expressed in proliferating cells (Gocke et al., 2000) and participates in spindle formation during mitosis (Zhang et al., 2002). Depletion of NudC leads to the occurrence of multiple spindles during metaphase and induces lagging chromosomes during anaphase (Zhou et al., 2003). Either depletion or overexpression of NudC components induces cytokinesis defects in mammalian cells (Aumais et al., 2003; Zhou et al., 2003). NudC is also intimately involved in the regulation of actin dynamics by stabilizing cofilin 1, a key regulator of actin polymerization and depolymerization (Zhang et al., 2016).

The NudC homologs possess a core CS domain (a domain shared by CHORD-containing proteins and SGT1) that occurs preferably in proteins with chaperone or co-chaperone activities (Lee et al., 2004; Zheng et al., 2011). The CS domain in the NudC family has a molecular architecture similar to small heat shock chaperones, such as p23 and HSP20/alpha-crystallin proteins (Garcia-Ranea et al., 2002). One of the most important functions of p23 and HSP20 is to simultaneously interact with HSP90 and specific client proteins (Lee et al., 2004; Botër et al., 2007). The CS domain is considered as a binding module for HSP90, implying that CS domain-containing proteins are involved in recruiting heat shock proteins (HSPs) to multiprotein complexes (Lee et al., 2004). Experimental evidences support both the HSP90 co-chaperone and intrinsic chaperone functions of the NudC family. For instance, the microtubule-associated *C. elegans* NudC homolog, NUD-1, has shown chaperone activity *in vitro*, inhibiting heat-induced aggregation and precipitation of citrate synthase and luciferase (Faircloth et al., 2009). Human NudC stabilizes Lis1 (lissencephaly protein 1) through HSP90-mediated pathways and exhibits intrinsic chaperone activity *in vitro* preventing aggregation of citrate synthase (Yang et al., 2010; Zhu et al., 2010). There have been only few studies aimed at exploring the functions of NudC proteins in plants (Jurkuta et al., 2009; Perez et al., 2009; Ishibashi et al., 2012; Silverblatt-Buser et al., 2018; Liu et al., 2019). In *A. thaliana*, a gene named *BOBBER1* (*BOB1*), encodes a small 34.5 kDa protein that interacts genetically with two 26S proteasome subunits and maintains a functional proteostasis network enabling proper plant development (Silverblatt-Buser et al., 2018). A partial loss-of-function mutation in *BOB1* results in numerous developmental defects, including obstructed root growth (Perez et al., 2009). BOB1 displays features typical for canonical small heat shock proteins (sHSPs), such as *in vitro* chaperone activity, heat stress induction and the availability of an α-crystallin-like NudC domain (Haslbeck et al., 2005; Perez et al., 2009). At high temperatures, BOB1 colocalizes with HSP17.6 and incorporates into heat shock granules that are formed mainly in the cytoplasm of heat-exposed plant cells (Kirschner et al., 2000; Miroshnichenko et al., 2005). It has been demonstrated that heat exposure of a partial loss-of-function mutant *bob1-3* during germination and at seedling stage leads to reduced thermotolerance, compared to wild-type seeds. This defect can be complemented by a functional *BOB1* transgene, confirming that the phenotype is a result of the reduced BOB1 function (Perez et al., 2009).

In search of regulators of root development, we identified a previously undescribed *Arabidopsis* homolog of the *Aspergillus NudC* gene, named *NMig1* (for *Nuclear Migration 1*), which shares structural similarity to other plant and mammalian *NudC-like* members. Since *NMig1* is a *BOB1* homolog and given the presence of the CS domain, we hypothesized the putative role of *NMig1* in root development and modulation of plant defense systems under stress. Accordingly, we generated *Arabidopsis* plants overexpressing *NMig1* and compared their root phenotypic features and responses to abiotic stress conditions (heat shock, drought, and high salinity) with those in the wild-type plants. Evaluation of the antioxidant status, accumulation of reactive oxygen species (ROS) and lipid peroxidation, as well as analysis of the expression levels of stress-related genes were undertaken. The results obtained strongly suggest a potential regulatory function of *NMig1* in root development and tolerance to abiotic stress.

## Materials and Methods

### Bioinformatics Analysis

The *NMig1* (*At5g58740*) sequence was used as a query to search for *NMig1* homologs from the NCBI database through BLAST analysis. The amino acid sequence of *NMig1* and some of its homologs were aligned using Clustal Omega (Pundir et al., 2016), and the alignment image was generated using BoxShade version 3.21 (K. Hofmann, Koeln, Germany and M. Baron, Surrey, United Kingdom). The accession numbers are as follows: Q8VXX3, NP_200682.1 (*Arabidopsis thaliana*); Q9LV09, NP_200152.1 (*A. thaliana*); Q9STN7, NP_194518.1 (*A. thaliana*); NP_001357629 (*Zea mays*); XP_002467510.1 (*Sorghum bicolor*); XP_015617572.1 (*Oryza sativa*); XP_003579589.1 (*Brachypodium distachyon*); NP_001236519.1 (*Glycine max*); XP_002525640.1 (*Ricinus communis*); XP_002274773.1 (*Vitis vinifera*), and CAL37614 (*Homo sapiens*). The conserved motifs and domain structure of NMig1 protein were analyzed using InterPro scan program^1^ (Supplementary Figure S1).

### Generation of Constructs and Transformation of *Arabidopsis thaliana*

The recombinant plasmids were obtained through the GATEWAY^TM^ recombination cloning technology (Invitrogen Life Technologies). The Entry Clones were generated by inserting the coding sequence of *NMig1* into the pDONR221 donor vector, then the Entry Clones were recombined into the destination vectors pK7WG2 or pK7FWG2 [with C-terminal translational green fluorescent protein (GFP) fusion] under the control of *CaMV 35S* promoter and *neomycin phosphotransferase II* (*nptII*) gene for plant selection (Karimi et al., 2007). The generated *35S:NMig1* and *35S:NMig1-GFP* constructs were introduced into *Agrobacterium tumefaciens* strain C58C1 (pMP90), harboring the helper plasmid *pSoup*. The positive clones were selected on agar solidified YEB nutrient medium supplemented with 100 mg l^−1^ rifampicin, 100 mg l^−1^ spectinomycin and 50 mg l^−1^ gentamicin, and subsequently transformed into wild-type Col-0 plants by the floral-dip method (Clough and Bent, 1998). The primary transformants (T_0_) were selected on 50 mg l^−1^ kanamycin and their progeny genotyped with gene specific and *nptII* gene primers. The positive transformants were further tested by kanamycin selection and five representative transgenic lines (L1 – L5) were generated. All analyses were carried out with homozygous T_3_ seedlings.

### Plant Material and Growth Conditions

*Arabidopsis thaliana* seeds were surface sterilized, stratified for 2 days in the dark at 4°C, and grown on half-strength Murashige and Skoog (1/2MS) (Murashige and Skoog, 1962) medium (pH 5.7) solidified with 1.0% agar at 22°C. To evaluate the root phenotypes of wild-type and transgenic (T_3_ generation) plants, approximately 30–35 seeds from each independent transgenic line and controls were plated at least in triplicate and cultivated in a growth chamber under a 16-h light/8-h dark cycle and a light intensity of ∼150 μmol m^–2^ s^–1^. For all phenotypic analyses, plants were vertically grown on square plates (Greiner Labortechnik) for 10 days unless otherwise stated. For comparison of the root phenotype of transgenic and wild-type plants, *NMig1*-overexpressing lines were co-cultivated with the wild-type plants on the same agar plates divided into two equal parts. Each plate contained seedlings of one individual transgenic line and Col-0. To analyze responses to abiotic stress, 5-day-old seedlings were transferred to plates containing solid 1/2MS medium supplemented with or without 150 mM sorbitol or 150 mM NaCl and scanned every day. Heat stress was applied by exposure of 8-day-old seedlings to 42°C for 1 h and subsequently transferred into a growth chamber for 2 additional days of growth under optimal conditions. Untreated wild-type and transgenic plants were used as controls and these were grown at the same time as the individuals subjected to the stress treatments.

### Morphological Characterization of Roots

Plates were scanned every day with an Epson Perfection V850 Pro scanner. Root length was quantified using the image processing software ImageJ 1.48 (National Institutes of Health, Bethesda, MD, United States), and the visible lateral roots were counted and calculated per seedling. Non-emerged lateral root primordia were quantified on the roots of 10-day-old seedlings after root clearing (Malamy and Benfey, 1997). The stress-induced inhibition of primary root growth, formation of lateral root primordia and emerging lateral roots was calculated in percentages for each genotype as a difference between growth and branching under control and stress conditions. The density of lateral root formation events was quantified by dividing the total number of emerged lateral roots plus lateral root primordia by the primary root length, and expressed as lateral root formation events per cm of primary root. In each experiment, the root system of at least 30 plants was examined under an Olympus BX51 upright microscope with a differential interference contrast (DIC) and XC50 digital microscope camera.

### Confocal Microscopy and Image Analysis

Fluorescent images were acquired on an inverted laser scanning confocal microscope Zeiss LSM 710 using the ZEN software package (Carl Zeiss, Germany) after excitation by a 488 nm argon laser and detected using the bandpass 505–530 nm emission filter setting. Autofluorescence of photosynthetic pigments was observed after excitation with a 543 nm helium-neon (He-Ne) laser and detected using the bandpass 560–615 nm emission filter. The root apical meristem was imaged on an Andor Dragonfly spinning disk confocal system equipped with the Fusion software (Andor Technologies, Inc.) and iXon897 EMCCD camera at 488 nm laser excitation. Confocal images were processed with ImageJ1.52r or the CellTool software^2^ (Danovski et al., 2018).

### Determination of Free Radical Scavenging Activity and Antioxidant Status

Fresh plant material (200 mg) was frozen in liquid nitrogen, grinded to fine powder in TissueLyser LT (QIAGEN) and mixed with 500 μl ice-cold 80% ethanol. The samples were centrifuged at 10 000 rpm for 30 min at 4°C and the supernatants were used for determination of antiradical activity by 2,2′-diphenyl-1-picrylhydrazyl (DPPH) Radical Scavenging assay, according to Brand-Williams et al. (1995), and antioxidant capacity by Ferric Reducing Antioxidant Power (FRAP) assay, as decribed by Benzie and Strain (1999).

The antiradical activity of the plant extract (40 μl) was measured by mixing it with DPPH solution (960 μl) in methanol (6.10^–5^M). The absorbance of the reaction solution at 515 nm was determined after 30 min of incubation at room temperature in the dark. The rate of antiradical activity was calculated by Trolox standard curve and the results were represented as μmol Trolox g^–1^ FW.

The antioxidant capacity assay is based on the reduction of ferric tripyridyl-s-triazine (Fe^3+^-TPTZ) complex to the blue colored ferrous (Fe^2+^) form by the antioxidants present in the plant extract. Briefly, 50 μl of the supernatant was mixed with 1.5 ml of freshly prepared FRAP reagent (0.3 M acetate buffer, 0.01 M TPTZ in 0.04 M HCl and 0.02 M FeCl_3_.6H_2_O in 10:1:1 proportion) and 150 μl distilled water. The optical density was spectrophotometrically determined after 15 min at 593 nm. Antioxidant capacity was expressed as μmol FRAP g^–1^ FW.

### Measurement of Lipid Peroxidation and Hydrogen Peroxide Content

Fresh plant material (200 mg) was frozen in liquid nitrogen, grinded to fine powder in TissueLyser LT (QIAGEN) and mixed with 0.1% trichloroacetic acid (w/v) (TCA). The samples were centrifuged at 10 000 rpm for 30 min at 4°C and the supernatant was used for determination of lipid peroxidation and H_2_O_2_ content.

Lipid peroxidation was determined by the thiobarbituric (TBA) method, which quantifies the amount of malondialdehyde (MDA) as the end product of the lipid peroxidation (Hodges et al., 1999). Reaction mixture contained 500 μl supernatant, 500 μl 0.1 M phosphate buffer (pH 7.3) ant 1 ml TBA (0.5% w/v). The samples were placed in a boiling water bath for 45 min. After cooling on ice, the sample absorbance was read at 440, 532, and 600 nm. The MDA extinction coefficient (ε = 155 mM^–1^ cm^–1^) was used for calculations of MDA concentration and expressed as nmol g^–1^ FW.

Hydrogen peroxide (H_2_O_2_) content was determined according to Wolff (1994). One ml of 0.1% TCA extract was mixed with 1.9 ml reagent (composed by 100 μM xylenol orange, 250 μM ammonium ferrous sulphate, and 100 mM sorbitol in 25 mM H_2_SO_4_). The absorbance of the reactions was evaluated spectrophotometrically at 560 nm after 30 min incubation at room temperature and H_2_O_2_ extinction coefficient used for the calculations was ε = 2.24 × 10^5^ M^–1^ cm^–1^.

### *In situ* ROS Assays

*In situ* detection of reactive oxygen species (ROS) accumulation was performed by nitroblue tetrazolium (NBT) and diaminobenzidine (DAB) staining methods, according to Kumar et al. (2014) with slight modifications, as described in Nguyen et al. (2017). For tissue-specific detection of the superoxide anion (O^2–^) abundance, 6-day-old *A. thaliana* plants grown on 1/2MS, were stained in freshly prepared 0.05% NBT (w/v) in 50 mM sodium phosphate buffer (pH 7.4) for 30 min, covered with aluminum foil and incubated on a lab shaker (80 rpm) at room temperature. To detect H_2_O_2_ content, the seedlings were stained for 1 h in 1 mg ml^−1^ DAB dissolved in 10 mM Na_2_HPO_4_ (freshly prepared). After the incubation in NBT and DAB solutions, the seedlings were transferred into bleaching solution (ethanol: acetic acid: glycerol in ratio 3:1:1) and kept at 4°C overnight. The analyzes of the samples were performed on the following day on an Olympus BX51 upright microscope coupled to an XC50 digital microscope camera.

### RNA Extraction and Gene Expression Analysis

Total RNA was extracted using the Trizol reagent (Invitrogen), followed by clean-up with RNeasy Mini Kit (Qiagen), including DNase I (Qiagen) treatment, according to the manufacturer’s protocols. The quantity and quality of RNA samples were evaluated with Thermo Fisher Scientific NanoDrop 1000 Spectrophotometer. Complementary DNA (cDNA) was synthesized from 1 mg of total RNA with the iScript cDNA Synthesis Kit (Bio-Rad), according to the manufacturer’s instructions. The quantitative PCR analysis was carried out in a BioRad CFX96 real time C1000 thermal cycler with SsoFast EvaGreen supermix (BioRad) or in a LightCycler 480 system (Roche Diagnostics) with the SYBR Green I Master kit (Roche Diagnostics), according to the manufacturer’s instructions. Conventional PCR analysis was performed with the same cDNA templates in a PCR Eppendorf Mastercycler (Eppendorf, Germany). Primer pairs were designed with Beacon Designer 4.0 (Premier Biosoft International) or Primer 3.0 (Untergasser et al., 2012). Efficiency curves were generated for each primer set and amplification efficiency between 98 and 102% was considered acceptable for a quantitative gene expression analysis (Raymaekers et al., 2009). Expression levels were normalized to those of *EF1*α and *CDKA1;1*. The average Cycle threshold (Ct) values of the reference genes *EF1*α and *CDKA1;1* were calculated using three untreated biological replicates. Delta Ct (ΔCt) values were obtained by subtracting from the average reference Ct values the individual Ct values of each treated and untreated sample within a specific gene dataset. The relative quantity (RQ = 2^ΔCt) and the normalization factor [NF = RQ (*EF1*α + *CDKA1;1*)] were used to calculate the normalized expression of the genes of interest. In semi-quantitative analyses, *EF1*α was used as a reference gene. Each individual sample was analyzed in triplicate. The list of specific primer sets for the monitored genes is provided in Supplementary Table S1.

### Statistical Analysis

All values reported in this study are the means of at least three independent experiments with three replicates, unless otherwise stated. Mean data from all plants on a single plate were used to generate a single replicate. The significance of the results and statistical differences were analyzed using Statgraphics Plus 5.1 software (Statistical Graphics, Warrenton, VA, United States). The data were evaluated by multifactor analysis of variance and expressed as mean ± standard error (SE). A *p*-value equal to or lower than 0.05 was considered statistically significant.

### Accession Numbers

The Arabidopsis Information Resource (TAIR) locus identifiers for the genes mentioned in this study are *At5g58740* for *NMig1*, *At5g53400* for *BOB1*, *At4g27890* for *BOB2*, *At3g53990* for *USP17*, *At4g27320* for *USP21*, *At1g08830* for *SOD1*, *At1g20630* for *CAT1*, *At4g35090* for *CAT2*, *At1g07890* for *APX1*, *At2g33210* for *HSP60*, *At4g24190* for *HSP90*, *At1g74310* for *HSP101*, *At5g52310* for *RD29A*, *At2g01980* for *SOS1*, *At1g07940* for *EF1a*, and *At3g48750* for *CDKA1;1*.

## Results

### NMig1 Is a Small HSP With NudC Domain

Proteins belonging to the NudC family contain a highly conserved NudC domain of approximately 140 amino acids that share significant sequence similarity in different species (Supplementary Figure S1). The *A. thaliana NMig1* (locus *At5g58740*) gene is located in chromosome 5 (Chr5: 23725675-23727628), and consists of five exons and four introns. The deduced protein sequence (accession number AED97093.1) is annotated in the databases as a “HSP20-like chaperones superfamily protein.” The coding sequence of *NMig1* is 477 bp in length and encodes a protein of 158 amino acids with a molecular mass of 18.2 kDa (pI 5.77) (Aramemnon database; Schwacke et al., 2003).

According to the *Arabidopsis* developmental and root maps in the eFP Browser (electronic Fluorescent Pictograph^3^), *NMig1* is expressed in an organ specific manner (Supplementary Figure S2). The developmental map showed that the highest expression level (expression potential) in the leaves, stem, flowers, pollen and seeds was about 510 absolute signal value (a.s.v.) (Supplementary Figure S2A). In the root, the *NMig1* expression potential reached about 2766 a.s.v. depending on the growth conditions. At tissue resolution within the root, the highest level of *NMig1* expression was found in the lateral root cap and columella cells (Supplementary Figure S2B).

In the *A. thaliana* genome, besides *NMig1*, two additional genes encode proteins with NudC domain, *BOB1* (*At5g53400*) and *BOB2* (*At4g27890*) (Supplementary Figure S1; Jurkuta et al., 2009; Perez et al., 2009). BOB1 and BOB2 show weak sequence similarity to NMig1. BOB1 displays 42.1% similarity, 27.3% identity, gaps 48.6% and overlap in 40 amino acids when compared to NMig1. BOB2 has 41.4% similarity, 30.7% identity, gaps 47% and overlap in 42 amino acids [The European Bioinformatics Institute, Pairwise Sequence Alignment, accession numbers: AAO23639.1 (BOB1), AEE85405.1 (BOB2)].

In addition, protein sequence analysis revealed the presence of a CS (CHORD-containing proteins and SGT1) domain (PF04969) that is typical for proteins functioning as independent sHSPs or co-chaperones of other HSPs (Zheng et al., 2011). The CS domain encompasses almost all of NMig1 protein and consists of approximately 100 amino acids. Comparison of NudC family members in different species displayed conservation of CS domain sequences across monocots and dicots, and also human (Supplementary Figure S1).

### Expression Patterns and Root System Architecture of *NMig1* Overexpressors

**FIGURE 1:**
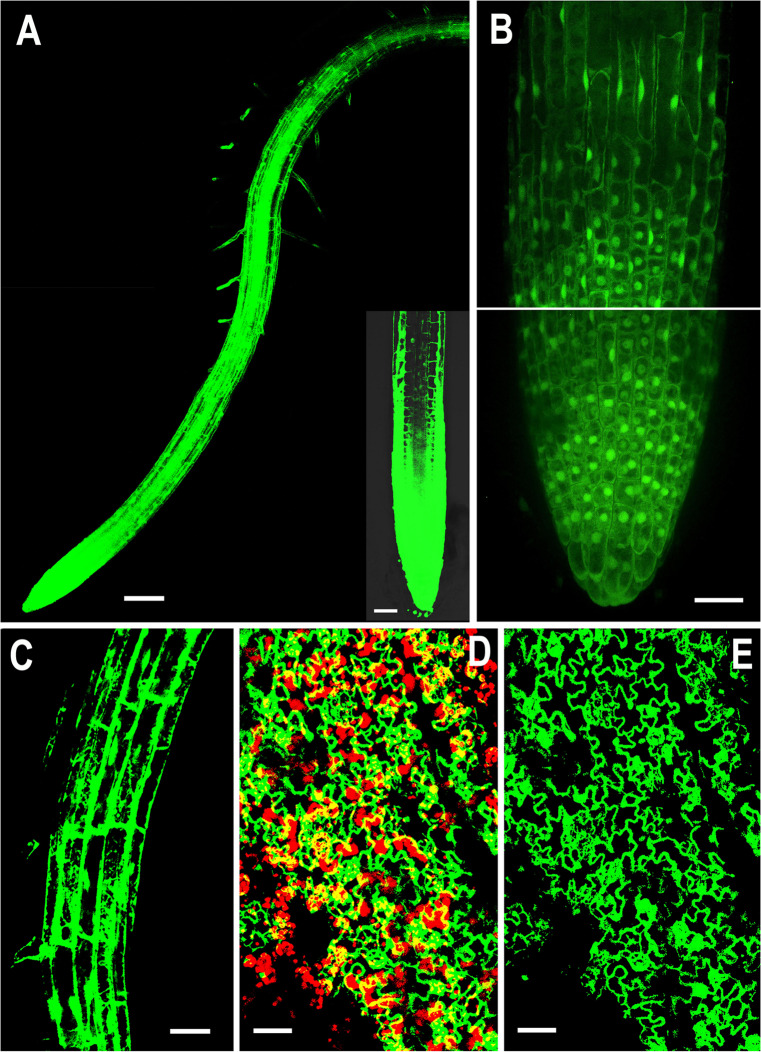
Representative confocal images showing localization of overexpressed *NMig1* gene in the root and leaf tissues of 6-day-old *Arabidopsis thaliana*. Expression pattern of *35S:NMig1-GFP* in: **(A)** primary root, **(B)** primary root meristem, **(C)** cells from root elongation zone, **(D)** and **(E)** leaf epidermal cells and chlorophyll autofluorescence (red). Scale bars: **(A)** 100 μm; **(B)**, **(D)** and **(E)** 20 μm; **(C)** 50 μm.

**FIGURE 2:**
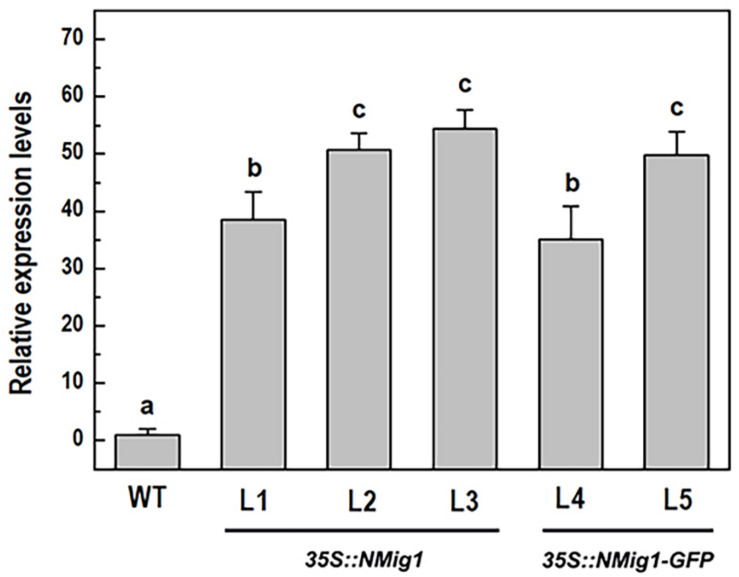
Expression analysis of the *NMig1* gene in 10-day-old wild-type seedlings and homozygous transgenic lines overexpressing *NMig1* (L1-L5). Expression levels were normalized to those of *EF1a* and *CDKA1;1*, as described in Materials and Methods. Data represents the mean values ± SE from at least three biological replicates. Different lowercase letters indicate statistically significant difference (*p* ≤ 0.05).

To investigate the biological functions of *NMig1*, we constructed five transgenic lines (L1, L2, L3, L4, and L5) that stably expressed *NMig1* under the control of *CaMV 35S* promoter. In lines L4 and L5, the *NMig1* gene was fused upstream of a reporter gene encoding the green fluorescent protein (GFP) (Figure 1). Quantification of *NMig1* expression in the generated homozygous lines revealed strong upregulation of *NMig1* transcripts, which increased between 35- and 50-fold compared to the wild-type values (Figure 2). Based on the obtained results, the lines with the highest *NMig1* expression levels (L2, L3 for *35S:NMig1* and L5 for *35S:NMig1-GFP*), were chosen for further phenotypic analyses and assessment of abiotic stress response.

Examination of the transgenic lines harboring the *35S:NMig1-GFP* construct demonstrated very strong expression in the primary roots, as the most intense signal was seen in the root apical meristem (Figure 1A). At the cellular level, high strength of the GFP signal was detected in the cytoplasm, in the nuclei, excluding the nucleolus (Figure 1B), as well as in the membrane and endomembrane systems (Figure 1C). Confocal imaging also showed intense GFP signal in the leaf epidermis, which was particularly strong in stomatal guard cells (Figures 1D,E). Overlap of *NMig1-GFP* and red chlorophyll autofluorescence in the leaf chloroplasts could be seen as yellow patches (Figure 1D).

**FIGURE 3:**
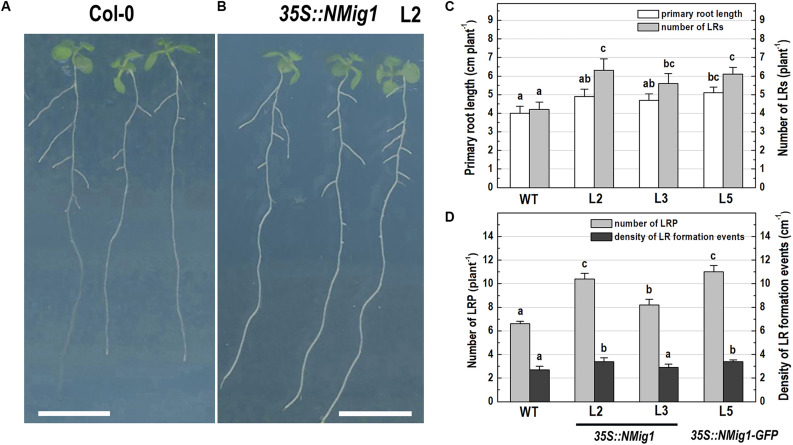
Primary root length and lateral root development of 10-day-old wild-type Col-0 seedlings **(A),** and transgenic lines overexpressing *NMig1* (L2, L3, and L5) **(B)** under optimal growth conditions: **(C)** average length of the primary root and number of lateral roots; **(D)** number of lateral root primordia and density of lateral root formation events. Different lowercase letters indicate statistically significant difference (*p* ≤ 0.05). Scale bar = 1.0 cm.

After 10 days of growth, a detailed phenotypic analysis was carried out to compare root growth and branching in the lines with ectopic expression of *NMig1* and the wild-type plants (Figures 3A–D). Under favorable growth conditions, all transgenic lines, overexpressing *NMig1*, displayed significantly greater primary root length (between 18 and 28%), as compared to the wild-type Col-0 (Figure 3C). The number of lateral roots was also enhanced, as the increase ranged between 33 and 50% in the different transgenic lines. To assess whether this increase was caused by enhanced lateral root initiation events, we additionally quantified the number of non-emerged lateral root primordia in the roots of *NMig1* overexpressors and the wild-type Col-0 (Figure 3D). The results showed an increased number of lateral root primordia (between 24 and 66%) in transgenic lines, as compared to the controls. Calculation of the density of all events associated with lateral root development that include emerged lateral roots and developing non-emerged primordia estimated as a function of primary root length, revealed 26% greater density of the formative events in L2 and L5, compared to the wild-type Col-0. In L3, despite that the number of all events associated with lateral root development increased about 30%, the calculated density did not show statistically significant difference from the control (Figure 3D).

### Differential Expression of *NMig1* Under Exposure to Abiotic Stress

**FIGURE 4:**
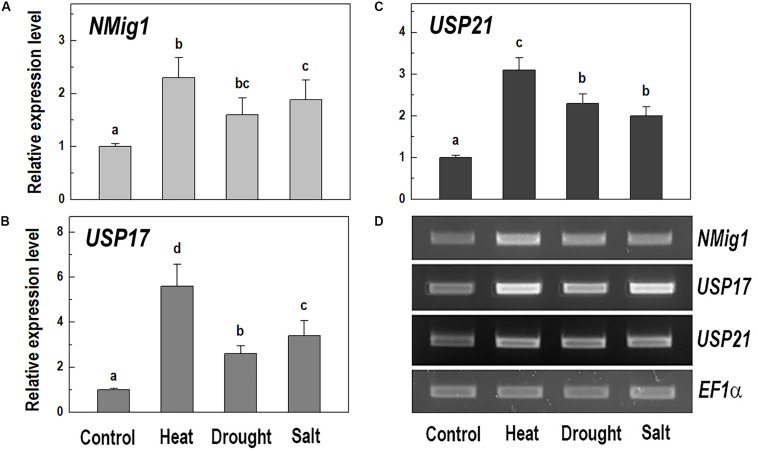
Quantitative RT-PCR **(A–C)** and semi-quantitative RT-PCR analysis **(D)** of the expression levels of *NMig1* **(A)**, and the universal stress proteins *USP17* **(B)**, and *USP21* **(C)** in 10-day-old wild-type seedlings subjected to heat shock, drought and high salinity. Expression levels were normalized to those of *EF1a* and *CDKA1;1*, as described in Materials and Methods. For semi-quantitative RT-PCR analysis *EF1*α was used as a reference gene. Data represents the mean values ± SE from at least three biological replicates. Different lowercase letters indicate statistically significant difference (*p* ≤ 0.05).

Given the presence of the CS domain, which has structural homology to the HSP90 co-chaperone p23 (Botër et al., 2007), we checked the possibility of involvement of NMig1 in abiotic stress responses. To study this experimentally, we quantified the *NMig1* transcript abundance after exposure of the wild-type Col-0 plants to heat shock, drought or high salinity (Figure 4). Expression of *NMig1* was significantly upregulated by all the applied stress treatments between 1.5- and 2.5-fold, compared to the control (Figure 4A). The degree of the *NMig1* stress induction was compared with that of two genes encoding universal stress proteins (USPs), *USP17* and *USP21*. The USPs are involved in a wide range of stress responses, and in general, their overexpression enhances stress tolerance and alleviates the oxidative damage through the activation of oxidative stress-responsive genes and the removal of intracellular ROS (Chi et al., 2019). Both *USP* genes studied were upregulated by the applied stress treatments (Figures 4B–D) with increases of 2.6- to 5.6-fold for *USP17* and 2.0- to 3.1-fold for *USP21*, compared to those of untreated controls. Thus, the stress-induced expression changes of *NMig1* were of similar magnitude and direction to those found for the studied USPs. The transcript abundance of the reference gene *EF1*α did not change under different experimental conditions (Figure 4D).

### Overexpression of *NMig1* Promotes Root Growth and Branching Under Abiotic Stress

**FIGURE 5:**
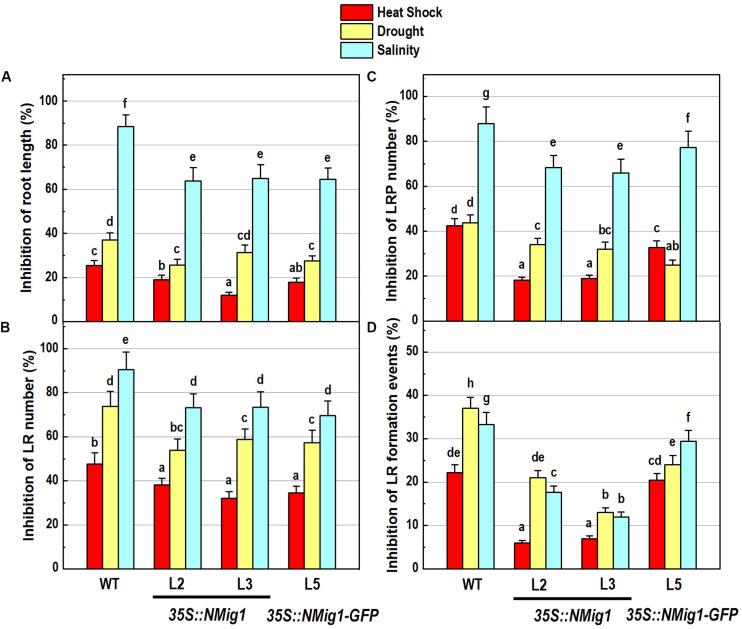
Inhibitory effect of various abiotic stress factors (heat shock, drought and salinity) on primary root length and lateral root development of 10-day-old wild-type seedlings and transgenic lines overexpressing *NMig1* (L2, L3, and L5): **(A)** inhibition of the primary root length, **(B)** inhibition of the number of lateral roots, **(C)** inhibition of the number of lateral root primordia, and **(D)** inhibition of the density of lateral root formation events. Different lowercase letters indicate statistically significant difference (*p* ≤ 0.05).

The response of transgenic plants to abiotic stress factors was analyzed in *NMig1* overexpressors and the wild-type Col-0 grown under the above-mentioned stress conditions, heat stress, drought and high salinity (Figure 5). Evaluation of the root system morphology when compared showed that all *NMig1*-overexpressing lines were more tolerant toward the tested abiotic factors since the stress-mediated reduction of root growth and branching was significantly lower, compared to the wild-type Col-0 (Figures 5A,B and Supplementary Figures S3A–L), as visualized by presenting the relative inhibition of the primary root growth and branching under each stress treatment within the tested genotypes (Figures 5A,B). The most obvious beneficial effect of the *NMig1* overexpression was seen for the number of lateral root primordia (Figure 5C) and density of all branching events under heat stress and salinity (Figure 5D). The heat- and salt-mediated reductions in these parameters were significantly lower in *NMig1*-overexpressing lines, compared to Col-0. Exposure to heat stress resulted in more than 20% inhibition in the density of all initiation and formative events associated with lateral root development in Col-0, whereas in the overexpressors L2 and L3, this parameter was inhibited by only about 6% (Figure 5D). Similarly, the inhibitory effect of drought and salinity on the density of all lateral root initiation events was at least twice lower in L2 and L3 than that observed in the wild-type.

### *NMig1* Overexpression Improves Free Radical Scavenging Activity and Antioxidant Status

**FIGURE 6:**
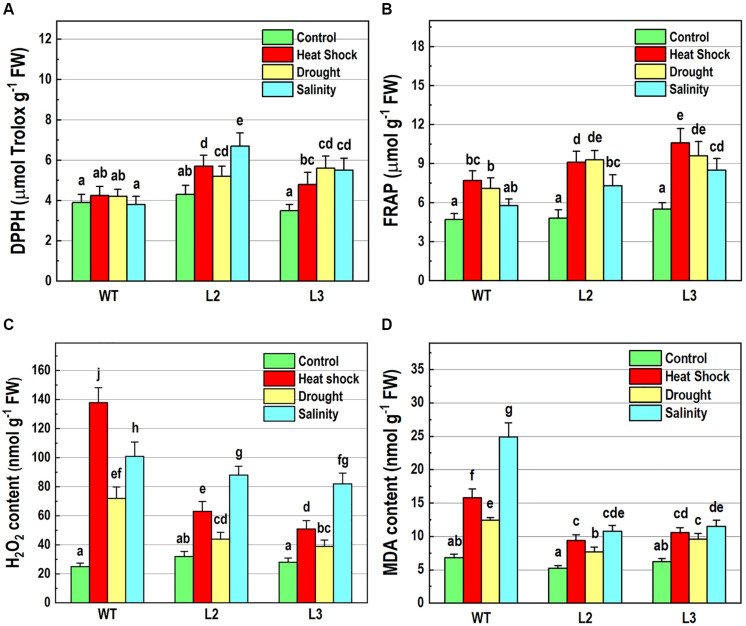
Determination of antioxidant-related parameters in 10-day-old wild-type seedlings and transgenic lines overexpressing *NMig1* (L2 and L3) after exposure to heat shock, drought and high salinity: **(A)** free radical scavenging activity, **(B)** antioxidant capacity, **(C)** hydrogen peroxide (H_2_O_2_) content, and **(D)** malondialdehyde (MDA) level. Different lowercase letters indicate statistically significant difference (*p* ≤ 0.05).

To further elucidate the abiotic stress responses of the transgenic plants, the effect of *NMig1* overexpression on the antioxidant status was monitored using two antioxidant assays, DPPH (Figure 6A) and FRAP (Figure 6B). Both analyses displayed that antioxidant status did not differ in unstressed transgenic and wild-type plants. The DPPH radical scavenging activity was increased by the overexpression of *NMig1* under all applied stress factors (Figure 6A). The assessment of antioxidant activity also showed higher stress-induced levels in the transgenic lines, as compared to the wild-type Col-0. Thus, both antioxidant assays showed similar trends of change manifested by lower values in the stress-treated wild-type seedlings and higher values in the transgenic lines (Figures 6A,B).

Hydrogen peroxide is one of the most common and early produced ROS in stress-treated plants and comparison of H_2_O_2_ content in *NMig1*-overexpressing and Col-0 plants displayed considerably reduced H_2_O_2_ production in the transgenic lines (Figure 6C). The most obvious difference between *NMig1*-overexpressors and Col-0 plants was observed under heat stress. Exposure of Col-0 to heat shock led to more than 2-fold higher H_2_O_2_ accumulation, compared to transgenic plants. Under salinity stress, the content of O2^•−^H_2_O_2_ greatly increased in both the wild-type Col-0 and *NMig1* overexpressors. However, this increase was less pronounced in the transgenic than in the wild-type seedlings.

Since ROS usually induce the peroxidation of membrane lipids, a quantitative assay was carried out to compare the level of lipid peroxidation, determined by the amount of MDA (Figure 6D). At control conditions, no significant difference in MDA content was detected between the *NMig1*-overexpressing and wild-type plants. However, the applied abiotic stresses induced greater accumulation of MDA in Col-0, as compared to that in *NMig1* overexpressors. Exposure to high salinity increased about 4.0-fold the content of MDA in Col-0 and less than 2.0-fold in the transgenic lines, compared to the respective controls. Similarly, under heat shock and drought *NMig1* overexpressors accumulated lower levels of MDA, compared to the wild-type Col-0.

**FIGURE 7:**
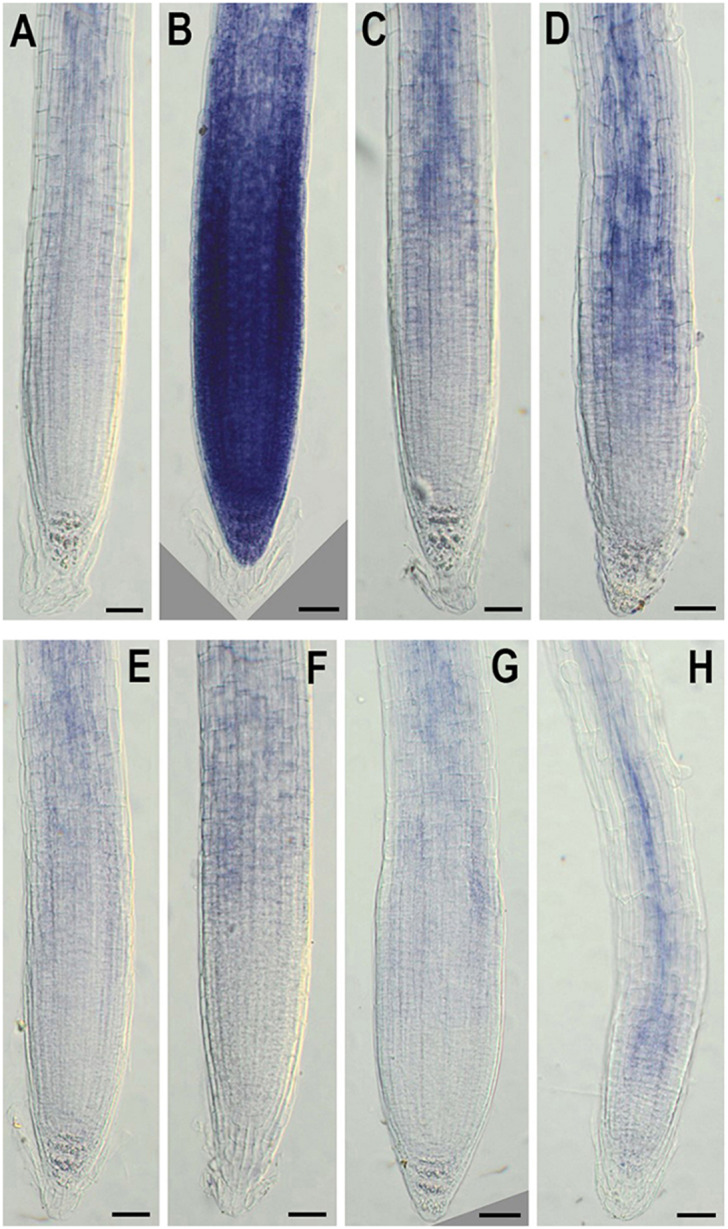
*In situ* detection of O2^•−^ by nitroblue tetrazolium (NBT) staining in 6-day-old wild-type **(A–D)** and *NMig1*-overexpressing **(E–H)** seedlings under optimal growth conditions **(A,E)**, and after exposure to heat shock **(B,F)**, drought **(C,G)** and high salinity **(D,H)**. Representative images from three independent experiments are shown. Scale bars = 50 μm.

**FIGURE 8:**
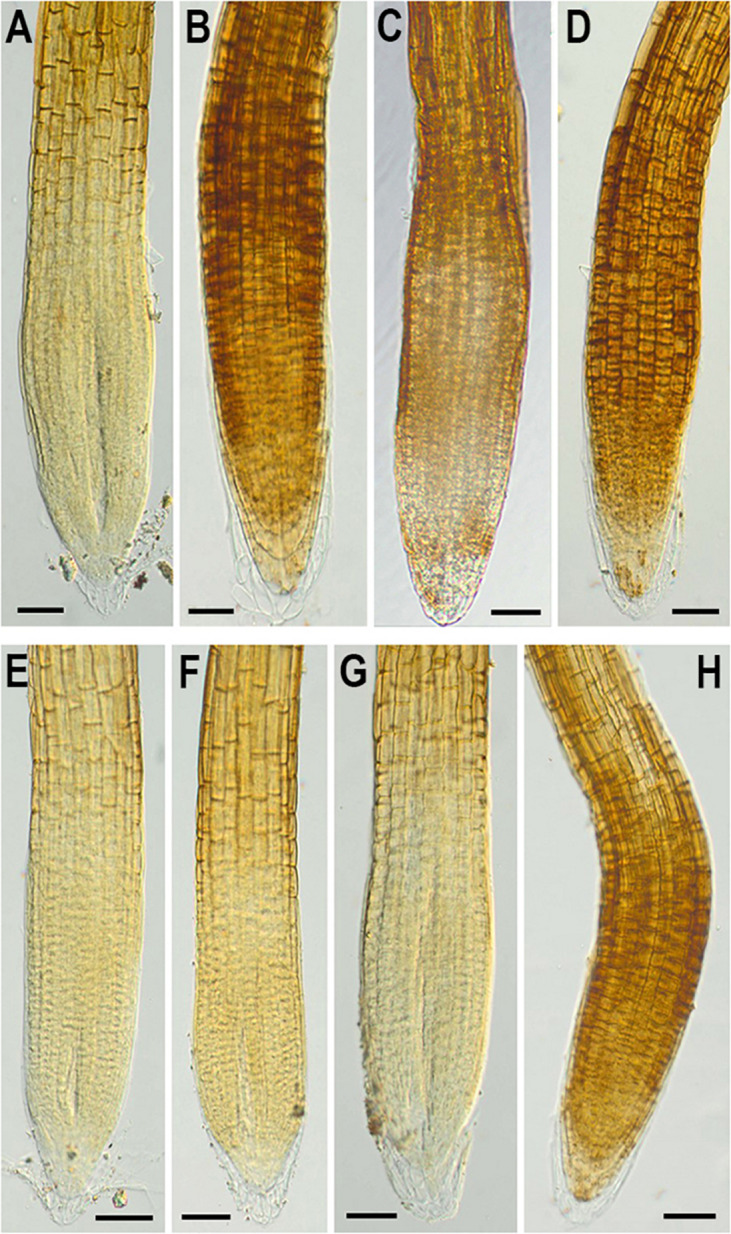
*In situ* detection of H_2_O_2_ by 3,3-diaminobenzidine (DAB) staining in 6-day-old wild-type **(A–D)** and *NMig1*-overexpressing **(E–H)** seedlings under optimal growth conditions **(A,E)**, and after exposure to heat shock **(B,F)**, drought **(C,G)**, and high salinity **(D,H)**. Representative images from three independent experiments are shown. Scale bars = 50 μm.

In addition, to evaluate the effect of *NMig1* overexpression on the stress-induced production of reactive oxygen species (ROS) in plant roots, the formation of superoxide anion (O2^•−^) and hydrogen peroxide (H_2_O_2_) was monitored using NBT (Figure 7) and DAB staining (Figure 8), respectively. Under benign growth conditions, both staining procedures showed that the O2^•−^ and H_2_O_2_ levels in transgenic and wild-type plants exhibited no differences (Figures 7A,E, 8A,E). Abiotic stresses, however, led to a higher accumulation of O2^•−^ and H_2_O_2_ in Col-0, where in the plant roots strong blue (Figures 7B–D) and brown (Figures 8B–D) staining was visualized. The most obvious difference between the *NMig1*-overexpressing plants and the wild-type Col-0 was detected under heat stress (Figures 7B,F, 8B,F). The accumulation of O2^•−^ in the heat-treated roots of Col-0 was very strong (Figure 7B), whereas in the *NMig1* overexpressors, the NBT staining intensity was compatible to the control level (Figure 7F). Exposure to drought and salinity also showed distinct staining patterns in Col-0 (Figures 7C,D, 8C,D) and in the transgenic plants (Figures 7G,H, 8G,H), but the difference in O2^•−^ and H_2_O_2_ contents between treated and untreated seedlings was considerably lower.

### Overexpression of *NMig1* Changed the Expression of Stress-Related Genes

**FIGURE 9:**
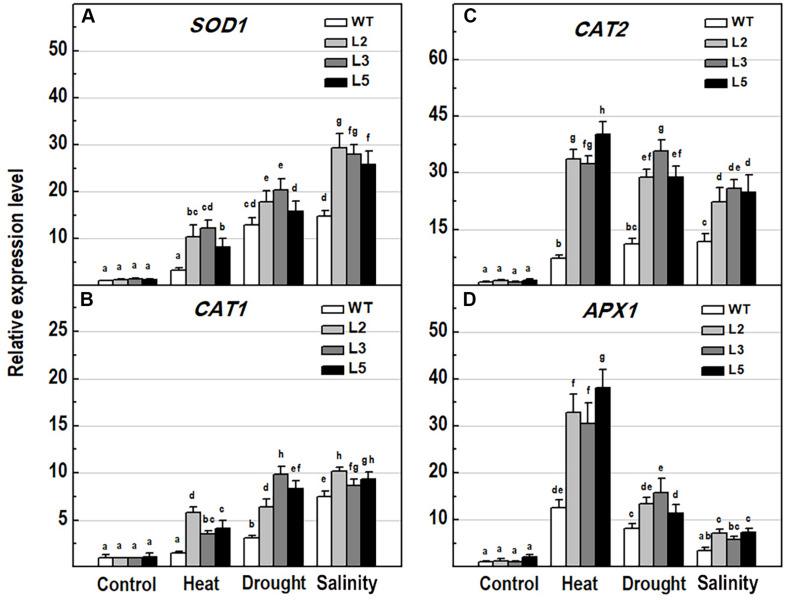
Analysis of the expression levels of major antioxidant genes in 10-day-old wild-type seedlings and transgenic lines overexpressing *NMig1* (L2, L3, and L5) after exposure to heat shock, drought and high salinity: **(A)** *SOD1*, **(B)** *CAT1*, **(C)** *CAT2*, and **(D)** *APX1*. Expression levels were normalized to those of *EF1a* and *CDKA1;1*, as described in section “Materials and Methods.” Data represents the mean values ± SE from at least three biological replicates. Different lowercase letters indicate statistically significant difference (*p* ≤ 0.05).

**FIGURE 10:**
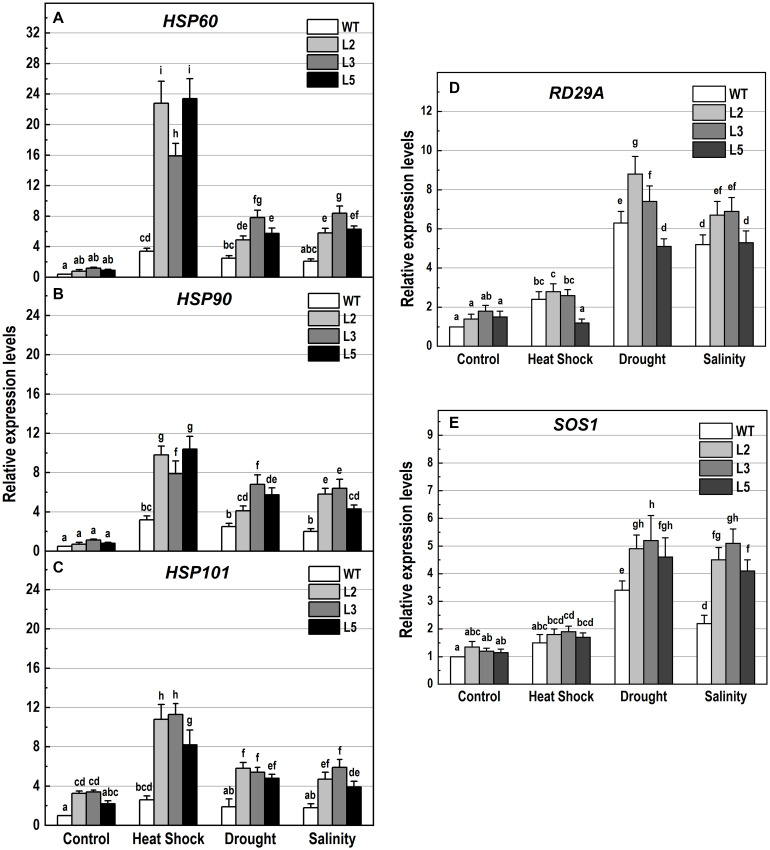
Analysis of the expression levels of genes encoding the heat shock proteins *HSP60*, *HSP90* and *HSP101*, and the stress-responsive genes *RD29A* and *SOS1* in 10-day-old wild-type seedlings and transgenic lines overexpressing *NMig1* (L2, L3, and L5) after exposure to heat shock, drought and high salinity: **(A)** *HSP60*, **(B)** *HSP90*, **(C)** *HSP101*, **(D)** *RD29A*, and **(E)** *SOS1*. Expression levels were normalized to those of *EF1a* and *CDKA1;1*, as described in section “Materials and Methods.” Data represents the mean values ± SE from at least three biological replicates. Different lowercase letters indicate statistically significant difference (*p* ≤ 0.05).

To explore the molecular mechanisms behind ROS detoxification under stress conditions, mediated by *NMig1* overexpression, we compared the expression levels of several genes encoding major enzymes involved in antioxidant defense (Figure 9) and other stress-related maker genes (Figure 10) in the transgenic plants and the wild-type Col-0. Very low expression of the genes *SOD1*, *CAT1*, *CAT2*, and *APX1*, encoding the ROS scavenging enzymes superoxide dismutase 1, catalase 1, catalase 2 and ascorbate peroxidase 1, respectively, was detected in the control plants (Figures 9A–D). The quantitative expression analysis revealed profound stress-induced upregulation of these genes in both the wild-type and *NMig1* overexpressors. More specifically, exposure of wild-type plants to heat shock, drought and salinity, increased the level of *SOD1* transcripts by 3.3-, 13-, and 14.8-fold, respectively, compared to untreated controls (Figure 9A). The expression levels of *CAT1* and *CAT2* also showed differential responses depending of the stress type (Figures 9B,C). In both the wild-type and *NMig1* overexpressors, *CAT1* was maximally induced by drought and salt stress (Figure 9B), while *CAT2* had the highest expression levels (more than 30-fold upregulation) under heat shock in the transgenic lines (Figure 9C). Similar expression pattern was observed for *APX1* after the plant exposure to heat shock, where the *APX1* expression increased by 12.5-fold in the wild-type but reached more than 25.0-fold in L2 and L3 (Figure 9D). When challenged with drought and salinity, the transgenic lines also showed higher accumulation of *APX1* transcripts, but the relative increase in the gene expression was lower, as compared to the control.

Additionally, the applied stress factors led to upregulation of genes encoding heat shock proteins, *HSP60*, *HSP90*, and *HSP101* (Figures 10A–C), and the abiotic stress-responsive genes *RD29A* and *SOS1* (Figures 10D,E). Exposure to stress of the wild-type plants resulted in an increase of all studied genes, however, this increase was much lower than that observed in the transgenic lines (Figures 10A–E). In *NMig1-*overexpressing plants, the heat stress induced greater accumulation of *HSP60* and *HSP101* transcripts, between 3.3-fold and 28.5-fold, compared to the transcript abundance in the same lines under benign growth conditions (Figures 10A,C). Sorbitol-mediated drought stress and high salinity led to an increase in the expression of *HSP60*, *RD29A* and *SOS1* between 3.3-fold and 7.25-fold, compared to the expression levels detected under optimal conditions.

## Discussion

In the present study, we identified a heretofore uncharacterized *Arabidopsis* gene, designated *NMig1* (for *Nuclear Migration 1*), which encodes NudC domain-containing protein. Overexpression of *NMig1* resulted in increased primary root growth and formation of more lateral branches, demonstrating that the studied root traits positively correlated with the higher level of *NMig1*. Consistent with these findings are the developmental defects including obstructed root growth due to a partial loss-of-function mutation in the NudC protein BOB1 (Perez et al., 2009). The stimulating effect on lateral root formation was nearly twice stronger than that on primary root growth. These results led us to further explore how overexpression of *NMig1* contributes to remodeling of root system architecture. Quantification of all branching events in the transgenic and the wild-type Col-0 plants indicated increased density of both emerging lateral roots and developing non-emerged primordia. Hence, NMig1 exerts a dual function in *Arabidopsis* root development, increasing lateral root initiation events and promoting lateral root emergence.

Generally, plants can adjust their root system in correlation with variable environmental conditions and it is very likely that every change in root architecture may contribute to plant performance under stress (Rellán-Álvarez et al., 2016; Motte and Beeckman, 2019). Overexpression of *NMig1* positively affected root growth and branching under heat shock, drought and salinity stress manifested by longer primary roots and more lateral branches in the transgenic plants compared to the wild-type Col-0. These beneficial root traits suggest that high expression levels of *NMig1* could at least partially counteract the inhibitory effect of different environmental extremes. In agreement with this observation is the transcriptional activation of *NMig1* upon exposure of the wild-type Col-0 to different stress factors. The level of *NMig1* induction by stress was compared to that of the genes encoding the universal stress proteins *USP17* and *USP21*. The USPs exhibit a redox-dependent chaperone function and are involved in plant protection from abiotic stress (Jung et al., 2015; Bhuria et al., 2016). The magnitude and profile of the stress-induced expression of both *USP* genes were similar to those of *NMig1*, which indicate that molecular adjustment of *Arabidopsis* to stress requires transcriptional activation of these three genes at comparable levels.

A common plant response to internal and external stimuli is the generation of ROS that can potentially lead to oxidative injury of cell components (You and Chan, 2015). Plant cells control the levels of ROS by balancing ROS generation and scavenging through antioxidant enzymes and molecules, such as superoxide dismutase, catalase, peroxidase and other antioxidants that protect cells from oxidative damage (Mittler, 2017). Under benign conditions, ROS are efficiently reduced by non-enzymatic and enzymatic antioxidant components, whereas under stress the production of ROS surpasses the capacity of the antioxidant defense systems to remove them, which causes oxidative stress (Vanderauwera et al., 2005). In our experiments, the expression of four genes, *SOD1*, *CAT1*, *CAT2*, and *APX1*, encoding major antioxidant enzymes was induced by the applied stress factors with especially high expression rates in the transgenic lines. This suggests that overexpression of *NMig1* largely upregulated antioxidant defense components in transgenic plants providing better protection against ROS damages. The overexpression of *NMig1* was also associated with greater total antioxidant activity and ROS scavenging potential, assessed by DPPH and FRAP assays. These observations correlate well with the reduced H_2_O_2_ levels in the stress-treated transgenic plants when compared with the wild-type Col-0. Additional histochemical detection of the accumulation of O2^•−^ and hydrogen peroxide (H_2_O_2_) specifically in the roots of the transgenic and wild-type seedlings also displayed a greater stress-induced staining in the wild-type Col-0, which was utmost under heat stress. In transgenic plants, a visible increase in staining intensities was not documented, in particular after the heat treatment. In addition, the degree of lipid peroxidation, a generally used marker for oxidative stress, quantified by the level of MDA concentration (Yadav et al., 2016) was considerably reduced in the transgenics compared to the wild-type.

Taken together, these results suggest that under stress conditions, different components of antioxidant defense system were mobilized by the overexpression of *NMig1*, which led to a decrease in ROS accumulation and reduction of oxidative damage in the transgenic plants. Hence, *NMig1* overexpressors possess a more efficient antioxidant system compared to the wild-type plants. Since the level of antioxidants in plant tissues is closely related to plant stress tolerance (Xing et al., 2007; Sofo et al., 2015), we can suggest that overexpression of *NMig1* confers tolerance to multiple abiotic stresses through optimization of antioxidant defense system (Zhang et al., 2013; Yong et al., 2017).

Furthermore, since *NMig1* encodes a HSP20-like chaperone superfamily protein, it could be expected that its overexpression is associated with increased accumulation of other HSPs. In general, various HSPs are coordinately expressed and operate in chaperone networks (Haslbeck et al., 2005; Waters, 2013). Many studies have shown a correlation between HSP induction and plant adaptation to stress (Jacob et al., 2017). We quantified the transcript levels of *HSP60*, *HSP90* and the well-known stress responsive gene *HSP101* (Queitsch et al., 2000) in the transgenic plants and Col-0 under abiotic stress. Although some expression differences depending on the nature of the stress imposed were detected, all tested HSPs followed a similar expression pattern, featuring substantial increase in *NMig1* overexpressors and lower detected levels in Col-0. Based on these results, we suggest that the *NMig1*-mediated induction of the studied *HSPs* could represent another mechanism contributing to abiotic stress tolerance in Arabidopsis. However, the exact molecular link between the HSP accumulation and protective role of *NMig1*-induced expression under various stress conditions still needs to be studied and defined.

It should be also noted that the impact of *NMig1* overexpression in promoting plant tolerance varied depending on the nature of the stress imposed. Exposure to heat shock coincided with the highest expression of the studied HSPs. Drought and high salinity led to a stronger induction of the stress-related marker genes *RD29A* and *SOS1* (Shi et al., 2000; Magwanga et al., 2019). This observation could be associated with the specific stress response mechanisms triggered by each particular stress factor and the involvement of *NMig1* in the concrete molecular relays. Nevertheless, overexpression of *NMig1* positively regulated the accumulation of defense gene transcripts, such as *HSPs*, *RD29A*, and *SOS1*, which is consistent with the higher stress tolerance of the transgenic plants.

In conclusion, the present work has provided novel insights into the involvement of plant NudC proteins in *Arabidopsis* root growth and in plant protection against abiotic stress factors. The proposed functions of NMig1 and the insufficient information on the precise biological roles of plant NudC proteins are challenging aspects that require further investigation.

## Data Availability Statement

The datasets generated for this study are available on request to the corresponding author.

## Author Contributions

VVa and TB designed the experiments and wrote the manuscript. VVe, IV, GZ, MZ, MG, NV, and VVa conducted the experiments and analyzed the data. All the authors read and approved the final manuscript.

## Conflict of Interest

The authors declare that the research was conducted in the absence of any commercial or financial relationships that could be construed as a potential conflict of interest.

## Footnotes

1 https://www.ebi.ac.uk/interpro/

2 dnarepair.bas.bg/software/CellTool

3 http://bar.utoronto.ca/efp/cgi-bin/efpWeb.cgi
