# Supplementary material for: Overexpression of the NMig1 Gene Encoding a NudC Domain Protein Enhances Root Growth and Abiotic Stress Tolerance in Arabidopsis thaliana
Source: Front Plant Sci. 2020 Jun 11;11:815. doi: 10.3389/fpls.2020.00815 (PMC7301909; doi:10.3389/fpls.2020.00815)
Supplement: Supplementary file 1 [file Image_1.pdf]

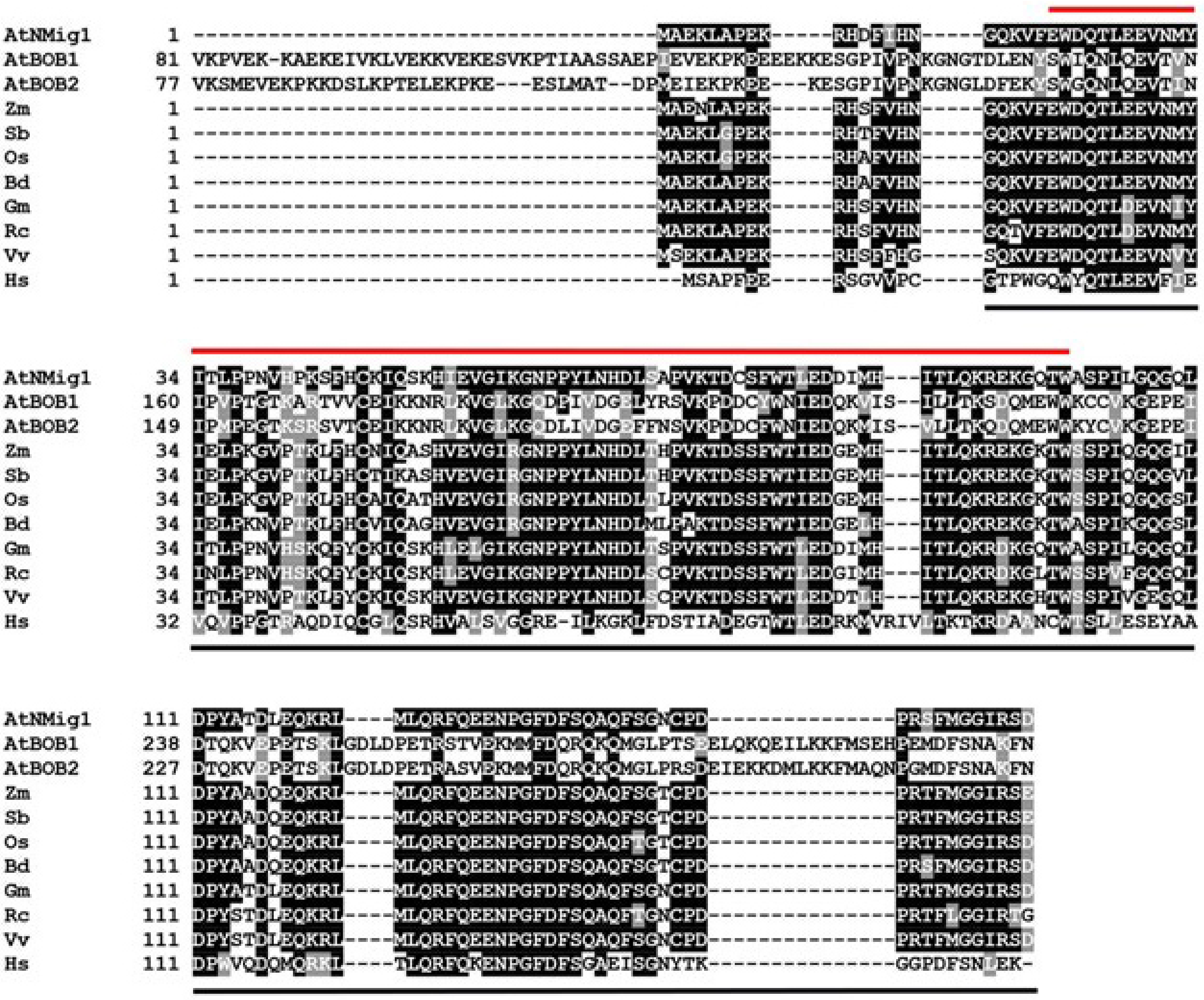

**At**, *Arabidopsis thaliana*; **Zm**, *Zea mays*; **Sb**, *Sorghum bicolor*; **Os**, *Oryza sativa*; **Bd**, *Brachypodium distachyon*; **Gm**, *Glycine max*; **Rc**, *Ricinus communis*; **Vv**, *Vitis vinifera*; **Hs**, *Homo sapiens*

FIGURE S1. Multiple alignment of amino acid sequences of the NudC domain proteins from Arabidopsis thaliana and their homologs generated by Clustal Omega. The presented species are *A. thaliana* (At), *Zea mays* (Zm), *Sorghum bicolor* (Sb), *Oryza sativa* (Os), *Brachypodium distachyon* (Bd), *Glycine max* (Gm), *Ricinus communis* (Rc), *Vitis vinifera* (Vv), and *Homo sapiens* (Hs). The two conserved domains are underlined with black (NudC domain) and red (CS domain) lines. Black background represents 90–100% sequence identity and grey background represents 80–90% identity.
