# Supplementary figures and images for: Overexpression of the NMig1 Gene Encoding a NudC Domain Protein Enhances Root Growth and Abiotic Stress Tolerance in Arabidopsis thaliana
Source: Front Plant Sci. 2020 Jun 11;11:815. doi: 10.3389/fpls.2020.00815 (PMC7301909; doi:10.3389/fpls.2020.00815)

At5g58740 247777\_at

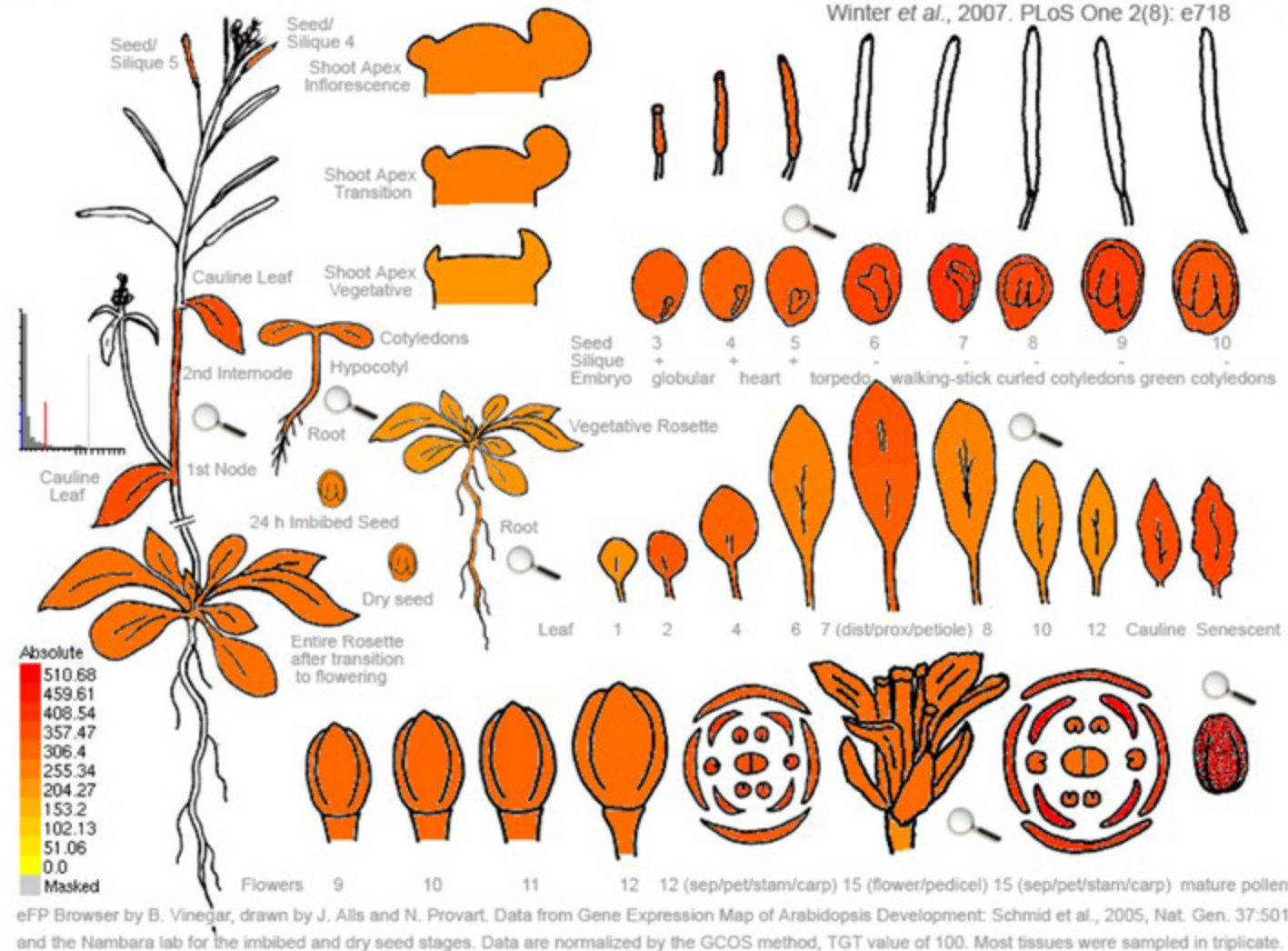

Absolute

|         |
|---------|
| 2766.45 |
| 2489.8  |
| 2213.16 |
| 1936.51 |
| 1659.87 |
| 1383.22 |
| 1106.58 |
| 829.93  |
| 553.29  |
| 276.64  |
| 0.0     |
| Masked  |

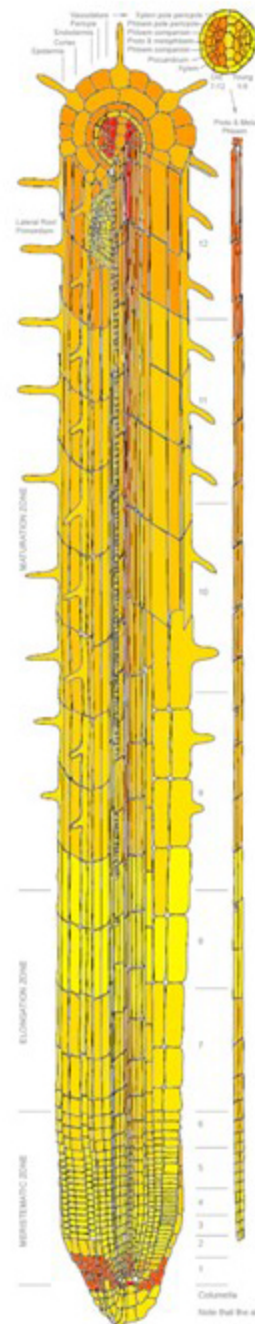

Supplement: Supplementary file 2 [file Image_2.pdf]
