# Supplementary material for: Overexpression of the NMig1 Gene Encoding a NudC Domain Protein Enhances Root Growth and Abiotic Stress Tolerance in Arabidopsis thaliana
Source: Front Plant Sci. 2020 Jun 11;11:815. doi: 10.3389/fpls.2020.00815 (PMC7301909; doi:10.3389/fpls.2020.00815)
Supplement: Supplementary file 3 [file Image_3.pdf]

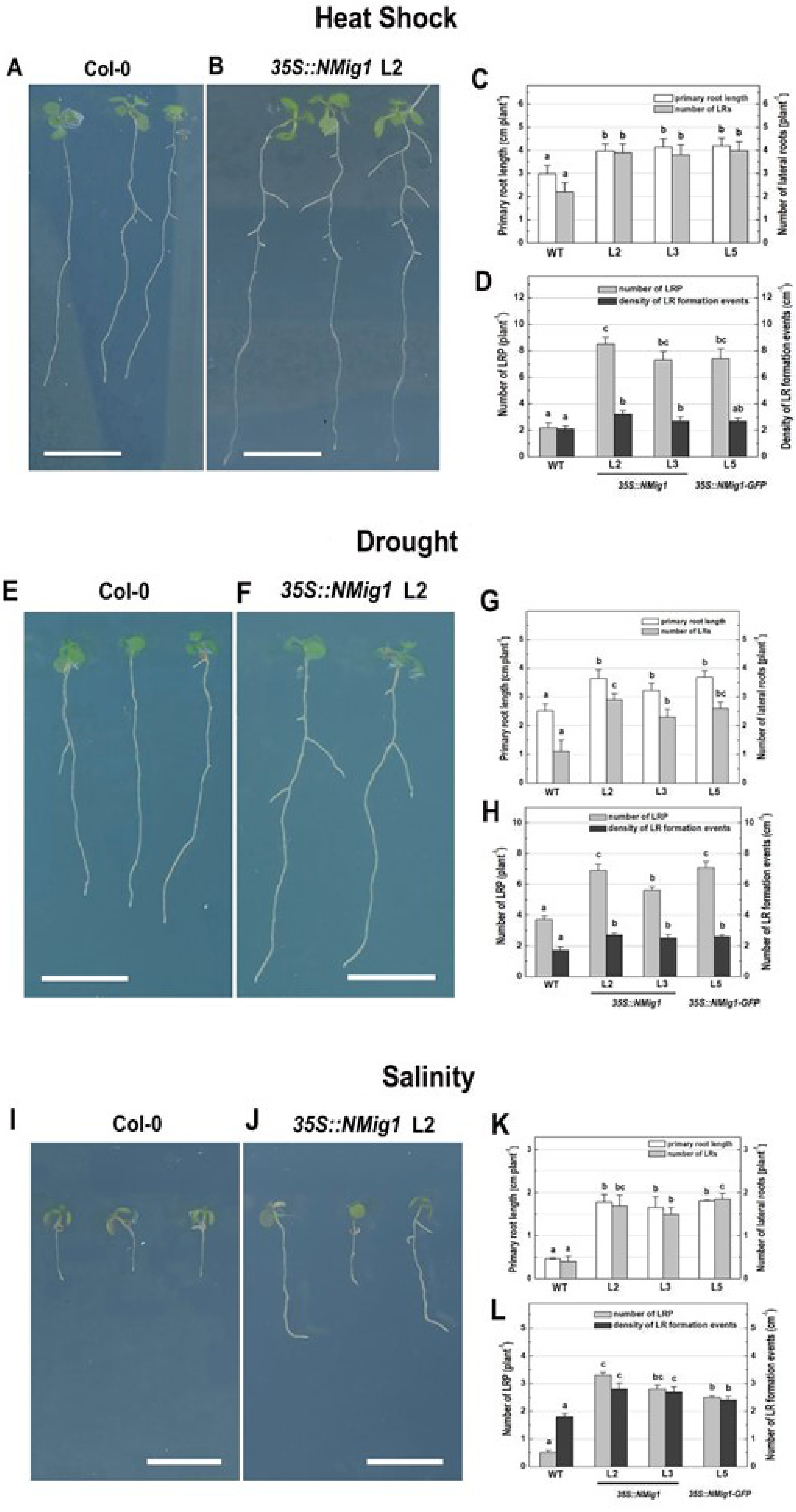

FIGURE S3. Phenotypic differences in primary root growth and branching between the wild-type Col-0 **(A,E,I)** and transgenic lines overexpressing *NMig1* **(B,F,J)** under heat shock induced by exposure to 42°C for 1 h **(A-D)**, drought stress induced by 150 mM sorbitol **(E-H)**, and high salinity induced by 150 mM NaCl **(I-L)**. The following root traits are evaluated: average length of the primary root and number of lateral roots **(C,G,K)**; number of lateral root primordia and density of all lateral root formation events **(D,H,L)**. The root traits are quantified in 10-day-old seedlings. Different lowercase letters indicate statistically significant difference (p≤0.05). Scale bar = 1.0 cm
