# Supplementary material for: Overexpression of the NMig1 Gene Encoding a NudC Domain Protein Enhances Root Growth and Abiotic Stress Tolerance in Arabidopsis thaliana
Source: Front Plant Sci. 2020 Jun 11;11:815. doi: 10.3389/fpls.2020.00815 (PMC7301909; doi:10.3389/fpls.2020.00815)
Supplement: Supplementary file 4 [file Table_1.pdf]

Supplementary Material

Supplementary Table S1. List of primer sequences used for RT-PCR analyses

Gene name	Locus	Amplicon length (bp)	Forward primer	Reverse primer
EF1a	*At1g07940*	156	ttgagatgcaccacgagtct	cctgggaggtgaagttagca
CDKA1;1	*At3g48750*	133	attgcgtattgccactctcatagg	tcctgacagggataccgaatgc
NMig1	*At5g58740*	157	tcattccactgcaaaatcca	tctccctcttctgcagggta
USP17	*At3g53990*	176	actggttcgaggcagaaaga	tggatcacaaagctgctcac
USP21	*At4g27320*	164	ttctccacgtttctccaacc	cgctagatccgctactttgg
SOD1	*At1g08830*	198	ggcgaaaggagttgcagttt	ccatcggggttgaaatgtgg
CAT1	*At1g20630*	193	gatctcggtatcccacagga	agagatctttggtggcatgg
CAT2	*At4g35090*	187	gagcctggagagagataccg	ctgctgagacttgaagaacgag
APX1	*At1g07890*	170	cctcgaaacaaggtatgacgga	taataacgccgatcgcagca
HSP60	*At2g33210*	201	caaagttggggtgctcctaa	ggcaactgatttgcatcctt
HSP90	*At4g24190*	186	tcaacccacgacaccctatc	tgttcagaccgctcttgact
HSP101	*At1g74310*	234	ggtcgatggatgcagctaat	ctctgtcttggattcgcaca
RD29A	*At5g52310*	224	gaacactccggtctctctgc	caatctccggtactcctcca
SOS1	*At2g01980*	223	gacgggagaatcaatcgaaa	tgctcttgctctcgtctcaa
